# The Tobacco-Free Village Program: Helping Rural Areas Implement and Achieve Goals of Tobacco Control Policies in India

**DOI:** 10.9745/GHSP-D-17-00064

**Published:** 2017-09-27

**Authors:** Nilesh Chatterjee, Deepak Patil, Rajashree Kadam, Genevie Fernandes

**Affiliations:** a Salaam Mumbai Foundation, Mumbai, India.; b University of Edinburgh, Edinburgh, Scotland, United Kingdom.

## Abstract

India has 274 million tobacco users and a tobacco use prevalence of 38% in rural areas. Tobacco consumption causes 1 million deaths and costs the health system nearly US$23 billion annually. Tobacco control policies exist but lack proper implementation. In this article, we review the Tobacco-free Village (TfV) program conducted in Maharashtra state in India and describe its process to help villages in rural India achieve “tobacco-free” status (i.e., the sale and use of tobacco are prohibited by law). We reviewed program documents and conducted 22 qualitative interviews with program staff and village-level stakeholders. From 2008 to 2014, Salaam Mumbai Foundation implemented the TfV program in 60 villages in Maharashtra state. The program used a number of strategies to help villages become tobacco free, including collaborating with a community-based organization, leveraging existing health workers, conducting a situation analysis, training health workers, engaging stakeholders, developing TfV assessment criteria, mobilizing the community, conducting health education, imposing sanctions, and offering incentives. By 2014, 4 villages had achieved tobacco-free status according to 11 assessment criteria. Successful villages demonstrated strong local leader involvement, ownership of the program, and commitment to the cause by residents. The TfV program faced barriers including poor motivation of health workers, difficulty in changing social norms of tobacco use, and refusal of local vendors to stop tobacco sales due to financial losses. This low-cost, community-driven program holds promise for helping public health practitioners and governments implement and achieve the goals of tobacco control policies, especially in resource-scarce settings.

## Abstract (precis)

Tobacco control and prevention in rural areas are possible as demonstrated by a community-driven tobacco-free village program in India. Success factors included community ownership with supportive program guidance, motivated and committed local leaders, collaboration with grassroots organizations, rewards and sanctions to establish new social norms, and provision of other income-generating options for vendors who sell tobacco. While the program required time and dedicated effort and was not successful in all villages, it holds promise for helping to achieve the goals of tobacco control policies, especially in resource-scarce settings.

## BACKGROUND

With 274 million adults using some form of tobacco, India has the second largest number of tobacco users in the world after China.^1^^,^^2^ Tobacco consumption causes nearly 1 million deaths and costs the Indian health system US$23 billion annually.^3^^–^^5^ An estimated 5 million children in India are addicted to tobacco; 4 in 10 tobacco users start consuming tobacco before the age of 18 years.^6^ Nearly 38% of adults in rural India use some form of tobacco, especially smokeless products. Many view tobacco use as a traditional practice,^2^ and many believe that tobacco relieves stress and reduces oro-dental pain.^7^^,^^8^ Tobacco consumption is strongly associated with low socioeconomic status.^9^^–^^11^

Tobacco consumption causes nearly 1 million deaths and costs the Indian health system US$23 billion annually.

Following advocacy efforts at national and regional levels, the Indian government enacted the Cigarettes and Other Tobacco Products Act (COTPA) in 2003.^12^ The COTPA prohibits smoking in public places, advertisements of tobacco products, and sale of tobacco products to and by minors (less than 18 years old); bans the sale of tobacco products within 100 yards of all educational institutions; enforces a mandatory display of pictorial health warnings on tobacco-product packaging; and mandates testing of all tobacco products for their tar and nicotine content. In 2004, India became the eighth country in the world to ratify the World Health Organization's (WHO's) Framework Convention on Tobacco Control.^6^

Despite these strides in tobacco control policy, studies assessing the implementation of the WHO's Framework Convention on Tobacco Control via the COTPA in India found lack of compliance with almost every provision.^13^^,^^14^ In Maharashtra, 1 of the 5 major tobacco-producing states in the country where 1 in 3 adults and 15% of adolescents consume tobacco,^15^^,^^16^ an observational study revealed only partial compliance with COTPA provisions on smoking and sale of tobacco to minors.^17^

Studies found that India lacked compliance with almost every provision of WHO's Framework Convention on Tobacco Control via the country's Cigarettes and Other Tobacco Products Act.

In this article, we review a tobacco control program and describe its process to help villages in rural India achieve “tobacco-free” status (i.e., the sale and use of tobacco are prohibited by law). The aim of the Tobacco-free Village (TfV) program, implemented between 2008 and 2014, was to stop the sale and use of tobacco in villages in Chandrapur district, in rural Maharashtra state, in order to reduce tobacco-related morbidities and mortality.

## METHODS

We analyzed program documents and conducted 22 qualitative interviews to describe the TfV program process and identify success factors and barriers in implementing a tobacco-free program. The program documents included operational plans, annual reports, meeting minutes, quarterly and monthly visit reports, local village council resolutions, financial budgets, and expenditure reports. We conducted 22 qualitative interviews with purposively identified program managers and coordinators, health workers, and village-level stakeholders.

## TOBACCO-FREE VILLAGE PROGRAM PROCESS

### Rationale, Collaboration, and Coverage

The TfV program was an initiative of the Salaam Mumbai Foundation (SMF), the rural wing of the Salaam Bombay Foundation (SBF), a nonprofit organization. SBF works on life-skills development for tobacco prevention and cessation with children and adolescents from low-income municipal schools in the city of Mumbai (previously Bombay), capital of the state of Maharashtra in western India. Inspired by the success of the urban program, SBF started SMF in 2007 to implement a similar program with children and adolescents in other districts of the state, with a special emphasis on rural areas.^18^ SMF initially started the rural tobacco-prevention program only in schools but soon realized that to achieve its goal of tobacco-free children in rural areas, it was imperative to involve and engage communities. This realization led to the rationale for designing a tobacco-free intervention at the village level. The TfV process for achieving a tobacco-free village is illustrated in the flowchart (Figure).

**FIGURE:**
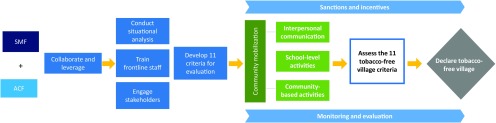
The Tobacco-Free Village Program Process Abbreviations: ACF, Ambuja Cement Foundation; SMF, Salaam Mumbai Foundation. Note: Many steps are iterative.

In 2008, SMF signed a formal collaborative agreement to implement the TfV program with Ambuja Cement Foundation (ACF), the corporate social responsibility division of a large cement manufacturing company in India. ACF works on livelihood programs related to health and agriculture in several districts in Maharashtra, including Chandrapur. ACF implements its health programs in villages via trained female health workers called *sakhis* (good friends), who conduct interpersonal counseling and referrals for government health services. SMF strategically implemented the TfV program in collaboration with an established organization like ACF to leverage ACF's grassroots presence in communities and its existing network of *sakhis*.

SMF initiated the TfV program in Chandrapur district in eastern Maharashtra, starting with 10 villages in 2008. The TfV program extended to 60 villages in Chandrapur district by 2014—specifically, 23, 22, and 15 villages in the 3 blocks (i.e., district subdivisions) of Rajura, Korpana, and Jiwati, respectively—serving a population of nearly 48,882 people in 11,375 households.

Between 2008 and 2014, the Tobacco-free Village program extended to 60 villages in Chandrapur district in Maharashtra state.

SMF spent 1.74 million Indian Rupees (INR) (approximately US$27,000) to implement the TfV program from 2008 to 2014; 75% was spent on program activities and the remainder on staff salaries in villages. Additional allocations of INR 500,000 (approximately US$7,800) on a part-time manager or coordinator at SMF and INR 245,000 (approximately US$3,800) on local and interdistrict travel were made over these 7 years. In the fiscal year April 2014 through March 2015, after ACF took over program implementation from SMF, it spent INR 300,000 (about US$4,700). Thus, between April 2008 and March 2015, the cost of implementing the program in 60 villages was approximately INR 2.8 million (about US$43,000). This amount does not include costs such as electricity, telecommunication, rent, and other organizational expenses.

### Tobacco-Related Situation Analysis

The first step of the TfV program was to conduct a situational analysis of tobacco consumption and socioeconomic factors in program villages in order to provide program planners with a contextual understanding to design appropriate change strategies. The findings of this analysis are summarized below.

The program villages had 683 residents, on average, with farming as the most common occupation, followed by work in coal mines and cement factories. Program villages had a total of 24 government schools that served grades 1 to 4 and 36 schools that served grades 1 to 7. Each village had more than 1 shop that sold tobacco. The villages were characterized by poor connectivity to district centers, with a transportation service frequency of 2 state-run buses each day. Few households owned televisions; *sakhis* used interpersonal communication and wall paintings as their main channels of communication.

Almost half of the men, women, and youth consumed tobacco in some form. Men generally used *kharra*, which is a mixture of tobacco, *chuna*, and cardamom (Table 1). Women consumed *kharra* and *mishri*, which is tobacco powder used for cleaning teeth; they put *mishri* in their mouths before going to work in the fields. Adolescents and youth consumed *gutkha* and *paan masala*, whereas elderly men preferred smoking *beedis*.

**TABLE 1.** Forms of Tobacco Consumed by Different Segments of the Rural Population, Chandrapur District, Maharashtra, India

Type of Tobacco	Description	Predominant Consumer
*Kharra*	A mixture of tobacco, areca nut, lime (*chuna*), and katechu (*katha*)	Men and women
*Mishri*	Powdered tobacco containing teeth-cleaning powder	Women
*Gutkha*	A powdery mixture of crushed areca nut, tobacco, katechu, paraffin wax, lime, and sweet or savory flavorings	Adolescents and youth
*Paan masala*	A mixture of betel leaf with lime, areca nut, clove, cardamom, mint, and tobacco, with other flavorings	Adolescents and youth
*Beedi*	A type of cigarette made of unprocessed tobacco flakes wrapped in tendu leaves (found widely across central India)	Elderly men

In the program villages, almost half of the men, women, and youth consumed tobacco in some form.

Unlike alcohol consumption, which carried a stigma, chewing tobacco was a culturally accepted practice that formed part of residents' daily routines. Several households served tobacco to guests as a welcoming gesture. Some families also held the belief that tobacco kills hunger pangs; they consumed it frequently during the day, especially when they could not get enough food.

### Training Existing Community Workers

SMF leveraged ACF's existing networks of *sakhis* to achieve the objectives of the TfV program in the program villages. For each village, ACF nominated 1 to 2 *sakhis* for program training in how to provide tobacco-related information and conduct community activities, including a step-by-step protocol for communication with households, with periodic supportive supervision.

The program trained *sakhis*, female health workers, to provide tobacco-related information and conduct community activities in villages.

### Stakeholder Involvement

After training the *sakhis*, the next step of the TfV program was to engage influential stakeholders from each village. A key stakeholder, the *sarpanch*, elected leader of the *gramsabha* (village governing council), generally has influence over the community. In Thutra village, for example, the *sarpanch* owned a shop that sold tobacco and initially refused to be a part of the TfV program. After SMF made several visits to discuss the issue with the *sarpanch,* he agreed to participate and ultimately closed his shop and became a champion for tobacco control in his village as well as neighboring areas.

Other influential stakeholders included members of the village council, local police officers, principals and teachers of local schools, and members of self-help groups, community-based organizations, and religious groups. SMF conducted an average of 30 meetings with village-level stakeholders every year to equip them with the information and skills needed to mobilize public support for tobacco control.

### Development of Assessment Criteria for Tobacco-Free Villages

The TfV program relied on a list of criteria for assessing the progress of villages in becoming tobacco-free. These criteria provided set targets and a road map to implement activities and monitor and evaluate the program in each village. SMF staff developed the criteria by holding meetings with stakeholders to discuss the provisions from the COTPA and then deliberating on which provisions to include and adapt as criteria for assessing tobacco-free villages. The final list included 11 criteria (Table 2).

**TABLE 2.** Criteria for Tobacco-Free Villages^a^

Criterion No.	Description of Criterion	Corresponding Section of COTPA or Guidelines for Tobacco-Free Educational Institutions
1	Signboards that say “No Smoking Area: Smoking and tobacco chewing are offenses here,” of 60 x 30 cm size, should be displayed at main public places.	Section 3 of the COTPA enforces warnings on signboards displayed prominently at the entrances of and inside public premises.
2	No smoking or chewing of tobacco inside the village by children, young people, men, women, aged, and other members/visitors.	Rule 3 of the Guidelines for Tobacco-Free Educational Institutions prohibits smoking or chewing of tobacco inside the premises of institutions.
3	Direct and indirect advertisements of tobacco products are banned in the village.	Section 5 of the COTPA prohibits advertisement of tobacco products.
4	Display of posters on harmful effects of tobacco at prominent points in the village.	Section 3 of the COTPA.
5	A copy of the COTPA shall be available with the head of village council and made available at any time for all villagers.	Rule 6 of the Guidelines for Tobacco-Free Educational Institutions requires a copy of the COTPA to be available from the head of the institution.
6	No sale and purchase of tobacco products inside the village and mandatory signage shall be displayed prominently near the main gate and on boundary wall of the village.	Rule 2 of the Guidelines for Tobacco-Free Educational Institutions prohibits the sale of tobacco products inside the premises of and within a radius of 100 yards from educational institutions; it requires mandatory signage in this regard to be displayed prominently near the main gate and on the boundary wall of the institution.
7	The village council should pass the resolution to implement the COTPA in the village and strictly follow its provisions.	Not applicable.
8	A tobacco control committee must be instituted in the village; it can be chaired by the village council head, and should include 2 members of the village council, a teacher, at least 2 parent representatives, a member of the school management committee and village council, and local police. The committee should monitor the tobacco control initiatives of the village, meet quarterly, and report to the block-level administration.	Rule 7 of the Guidelines for Tobacco-Free Educational Institutions requires the setting up of a tobacco control committee, which is chaired by the school head/principal and comprised of a school teacher and counsellor (if available), at least two students, at least two parent representatives, a local member of the legislative assembly, the Municipal Councillor, and members of community-based organizations. The committee is to monitor the tobacco control initiatives of the village, meet quarterly, and report to the district administration.
9	All prominent groups such as self-help groups, youth *mandals* (groups), NGOs, schools, women's groups, local committees, and school management committees should be tobacco free and take initiative to make the village tobacco free.	Emerged from discussion with village-level stakeholders.
10	Visitors/outsiders cannot carry tobacco products when they enter the village.	Emerged from discussion with village-level stakeholders.
11	The village council must take up tobacco-control initiatives, including following up on the TfV process during meetings and prohibiting visitors from consuming tobacco in the village.	Rule 8 of the Guidelines for Tobacco-Free Educational Institutions requires the integration of tobacco-control activities with the ongoing School Health Programme of the State.

Abbreviations: COTPA, Cigarettes and Other Tobacco Products Act; SMF, Salaam Mumbai Foundation; TfV, Tobacco-free Village.

^a^ Developed by Salaam Mumbai Foundation and village-level stakeholders, based on the COTPA (2003)^12^ and Guidelines for Tobacco-Free Educational Institutions (2009).^19^

The Tobacco-free Village program developed a list of 11 assessment criteria based on the Cigarettes and Other Tobacco Products Act of 2003.

For each village to be declared tobacco free, an external observer had to assess it and determine whether it successfully met all 11 criteria. The *sakhi*, *sarpanch*, or other villagers assigned to the task used these 11 criteria to monitor the program (Table 2).

### Community Education and Mobilization

The TfV program used multitiered communication channels for educating and mobilizing community members to participate in tobacco control and prevention activities in each village. *Sakhis* conducted interpersonal communication activities during home visits using various information, education, and communication materials such as flipbooks and posters to educate families about the harms of tobacco and how to quit using it. *Sakhis* also held group meetings with youth, adolescents, and pregnant women, and they trained women members of self-help groups, who then conducted educational sessions with different audiences in the village.

In schools, the program trained teachers to conduct classroom sessions on the harmful effects of tobacco use, and students learned how to refuse if an adult asked them to buy tobacco. Competitions—including drawing, essay writing, rallies, and poster campaigns—incentivized students to become involved in tobacco prevention efforts. The program helped schools form student groups called *Bal Panchayats* (child governing councils) to raise awareness about problems associated with tobacco use. Schools organized tobacco-prevention events around commonly celebrated religious festival days, as well as World No Tobacco Day and World Cancer Day, encouraging participation from families. School principals and staff were encouraged to use every possible opportunity to talk to parents about the harms caused by tobacco use. Parents attended anti-tobacco sessions in the schools, conducted by trained self-help groups.

*Sakhis* conducted home visits, held group meetings, and trained group members to conduct educational sessions in the villages.

The TfV program organized community-based activities, such as wall paintings, poster campaigns, street plays, and village rallies, to focus public attention on the tobacco problem. Since few households owned televisions, the program staff organized tobacco-prevention film screenings followed by group discussion every month. The films were entertaining and were considered stress relievers after hard days of work in the fields. More importantly, this medium was persuasive—it got villagers to consider tobacco-related behavior change. Community activities reinforced the interpersonal and school-level efforts for tobacco prevention and behavior change.

Tobacco-prevention film screenings were a persuasive medium to encourage villagers to change tobacco-related behavior.

Finally, program staff set up educational booths during village fairs and cultural festivals, where they spoke about the benefits of a life without tobacco. Stakeholders such as the *sarpanch*, village council members, and police officers attended such community-based events and encouraged villagers to quit tobacco use and choose a healthier life.

### Sanctions and Incentives for Change

The village governing councils in Mangi and Thutra passed resolutions that made the sale of tobacco punishable by law; these sanctions acted as a major facilitator in prohibiting the sale and use of tobacco in order to meet the criteria for becoming tobacco-free villages. Financial incentives in the form of a cash award of INR 100,000 (US$1,556) for outstanding work in tobacco control were awarded by SMF's parent organization, Narotam Sekhsaria Foundation. The *sarpanch* from Thutra village and a motivated *sakhi* from a non-TfV village each received the coveted cash award based on their approaches and efforts in tobacco control. The disbursal of financial awards to someone they knew motivated *sakhis* and stakeholders to amplify their tobacco control efforts; and helped push the tobacco control agenda further in the selected villages. These incentives also encouraged winners to intensify and sustain their work. While financial rewards were found to be helpful in the initial phase of the TfV program, the program had to include nonmonetary incentives to ensure sustainability, such as appreciating the efforts of *sakhis* during biannual meetings and public recognition in village meetings and events.

### Sustainability

Until 2011, ACF's network of *sakhis* served the program on a voluntary basis. After this year, SMF provided 5-year funding to ACF for implementing the TfV program, so ACF would not have to rely entirely on voluntary resources. *Sakhis* received financial compensation and program staff had higher budgets to conduct comprehensive activities. SMF integrated all TfV program activities into the ACF operational system for seamless implementation in the future. After the 5-year funding period ended, ACF continued to implement the TfV program with internal financing.

**Figure:**
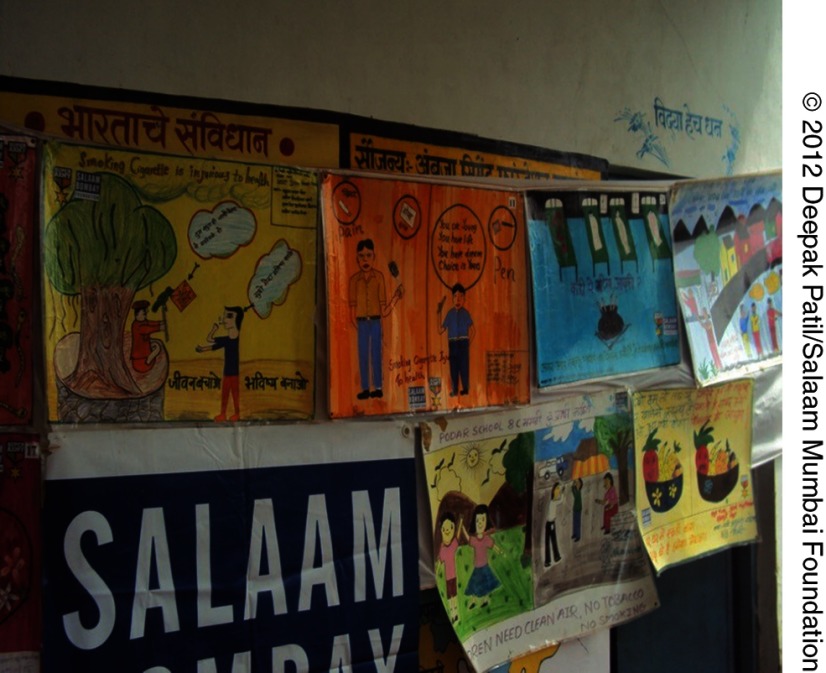
Tobacco control posters by students in a government school in the village of Mangi, India.

### Monitoring

Program staff and stakeholders conducted 3 levels of monitoring, using the 11 assessment criteria. At the first level, ACF staff, including *sakhis*, conducted day-to-day monitoring of program activities. At the second level, local school teachers and village-level stakeholders acted as watchdogs by periodically assessing the program status and providing feedback to the program staff. At the third level, senior SMF staff conducted annual observational visits and comprehensive monitoring of all program activities and adherence with the 11 tobacco-free village criteria. Additionally, ACF field teams sent monthly monitoring reports to SMF. Regular meetings also were held between the SMF and ACF program teams, as well as annual review meetings for the *sakhis*.

## CHALLENGES

### Community Norms

Addressing the long-standing tradition of tobacco use proved to be the greatest challenge for program staff. It was difficult to persuade villagers to stop using a product they had been consuming for years and that was embedded in their daily routines. It was considered normal for a child, adolescent, adult, or elderly person to consume tobacco. Furthermore, villagers had little or no information about the ill effects of tobacco consumption. In the 4 tobacco-free villages, influential leaders and stakeholders, positive role models, consistent discussions at all levels about the harms of tobacco, and sanctions changed the existing community norms around tobacco use.

Persuading villagers to abandon long-standing traditions of tobacco use was the greatest program challenge.

### Behavioral Constraints

At the start of the TfV program, *sakhis* were unable to successfully broach the topic of tobacco among households, as many of the *sakhis* were tobacco users themselves. As a result, villagers questioned their credibility as health workers counseling on the ill effects of tobacco. ACF addressed this behavioral barrier through an organizational initiative in which they provided *sakhis* with individual counseling and cessation support in the form of information about nicotine withdrawal symptoms and management techniques, oral health screenings, and reminders by supervisors to maintain positive behaviors. ACF also passed a no-tobacco policy that prohibited all program staff, including *sakhis*, from using tobacco. Although ACF conducted oral health screenings for villagers in some program villages, regular monitoring, follow-up, and cessation enforcement were not possible administratively.

*Sakhis* received support to stop using tobacco themselves, which improved their credibility as counselors on the ill effects of tobacco.

In villages where the *sarpanch*, village council member, or other important stakeholder owned a tobacco shop or used tobacco, stopping the sale and use of tobacco was difficult because people pointed to this leader as justification for their own tobacco use behavior. Even in villages where tobacco was prohibited, it was possible that some people went to neighboring villages to buy tobacco products and consumed it in the privacy of their homes.

### Economic Loss

A complete ban on the sale of tobacco products meant that shopkeepers would face losses in income, which made them unwilling to participate. Providing alternative means of income for tobacco vendors was challenging, as the sale of tobacco products was more lucrative than the sale of many other consumer products. Wherever tobacco vendors were reluctant to stop sales after many rounds of discussion, local police intervened. However, in some villages, local police officers refused to intervene because they had a connection with the vendors. In such cases, ACF and SMF program staff lobbied with higher-level officers in the district to intervene and stop tobacco sales.

### Programmatic Barriers

Training the *sakhis* in tobacco control was time-consuming and took almost a year. Since the *sakhis* did not receive monetary benefits for their work until 2011, they viewed TfV-related tasks as a burden beyond their regular duties, and they did not want to spend much time on them. Conducting regular follow-up visits to monitor and enforce tobacco-free status of the villages was therefore difficult. In the absence of financial compensation, these programmatic barriers were addressed by providing the *sakhis* with nonmonetary rewards and recognition during meetings and events. Another barrier was that any change in the team's composition affected program implementation. This challenge was addressed by integrating TfV processes into ACF's system such that any staff member could conduct all program activities seamlessly.

## LESSONS LEARNED

Beginning with 10 villages in 2008, the TfV program expanded to 60 villages by 2014. The TfV program declared the 4 villages of Thutra, Khairguda, Mangi, and Loldoha to be tobacco free according to the 11 program criteria. Although 2 other villages—Koolimadu^20^ and Gariphema^21^—had been declared tobacco free according to non-TfV program criteria, Thutra, Khairguda, Mangi, and Loldoha were the first to be declared tobacco free using the 11 criteria based on COTPA provisions.

The Tobacco-free Village program declared 4 villages tobacco free according to its criteria.

Why did some villages meet the TfV criteria while the overwhelming majority did not? We gained insights into the differences between these villages through interviews with village-level stakeholders such as the *sarpanch*, school principals, teachers, health workers, owners of shops previously selling tobacco, and police officials.

All the program villages, which were distributed across 3 blocks, had similar socioeconomic profiles, with farming and employment in coal mines and cement factories as the main sources of livelihood. Tobacco-free villages were found to be smaller compared with other villages, in terms of households (126 versus 194 on average) and population (531 versus 835 on average), although there was no significant statistical difference.

Motivated and committed local leadership stood out as the critical success factor for change in each of the 4 tobacco-free villages. Thutra village became tobacco free in 2012, and it was a motivated *sarpanch* who was responsible. In Khairguda village, which achieved TfV status in 2013, an inspired tobacco seller lit a symbolic bonfire of tobacco products from his shop, which made a persuasive case for others to join the movement. Mangi village also achieved tobacco-free status in 2013 after its local governing council passed a resolution and involved the police in stopping sale of tobacco. In Loldoha village, which became tobacco free in 2014, a committed *sakhi* worked adeptly through regular group meetings and door-to-door visits to inspire behavior change among villagers.

Motivated and committed local leadership stood out as the critical success factor for change in each of the 4 tobacco-free villages.

Achieving tobacco-free status has to be a process owned and led by the community. It must involve local leaders who are passionate and committed, have influence over people, and are willing to volunteer their time and efforts. Identifying, recruiting, and motivating the right leaders are the most important steps for achieving tobacco-free villages. This has to be followed by strengthening the capacity of leaders and stakeholders, providing supportive supervision of activities, offering feedback, and creating reward and recognition systems. This kind of a community-led model seems useful for tobacco control in resource-scarce settings; however, generating community ownership is an arduous process. For instance, bringing together local leaders and villagers to form a tobacco control committee required constant, consistent guidance and follow-up from program staff. Program planners need to sustain community participation in and ownership of activities, and enable families to champion the cause, rather than let the TfV program be perceived as an external agency's agenda.

Many villages that did not achieve tobacco-free status lacked the involvement of local leaders and community stakeholders, especially the village governing council, highlighting the need for strategic approaches to achieve buy-in and ownership from these influential community members. Many of the village governing council members are tobacco users themselves. There is also the issue of political patronage of tobacco vendors. The long-standing normative practice of tobacco use in rural India was another major obstacle to achieving tobacco-free status in the large majority of the villages. However, using a contextually appropriate, multitiered approach of community education and mobilization, advocacy with influential members including government officials, along with village-level sanctions, helped the TfV program to change norms in the 4 tobacco-free villages. Changing norms is difficult; it can take time. Therefore, an effective social and behavior change communication strategy at the state or national level has to be designed and implemented for tobacco control in rural India.

**Figure:**
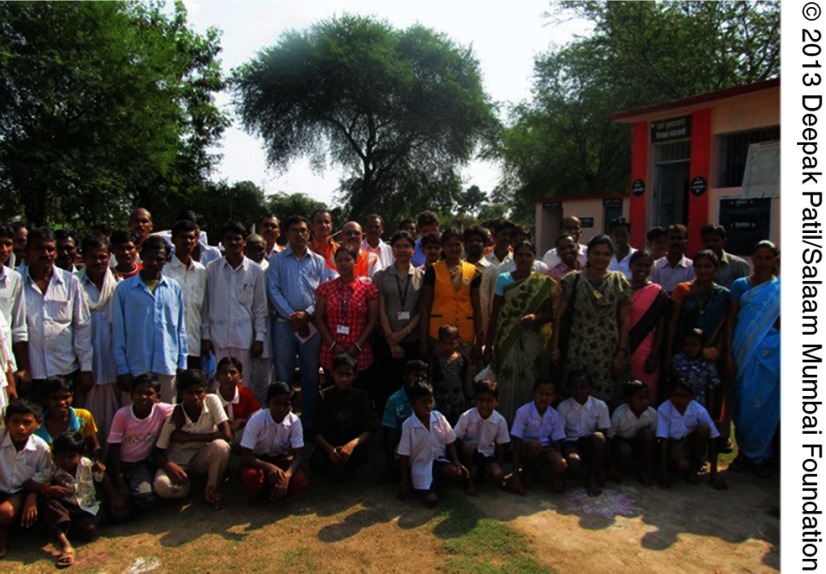
Program staff with *sakhis*, community members, and children in the village of Khairguda, India.

In 2016, the Public Health Department of Maharashtra circulated guidelines for setting up district-, block-, and village-level enforcement committees to ensure COTPA implementation.^22^ Ensuring tobacco-free villages requires more steps by the government: incentivizing village participation in the tobacco-free movement through rewards and recognition of TfVs as model villages; putting sanctions in place to dissuade noncompliant villages from continuing tobacco use; and partnering with established grassroots organizations to implement tobacco control and prevention activities in their program areas along with ongoing health and development projects.

One of the major lessons learned was that collaboration with grassroots organizations is critical for successful implementation and sustainability of the rural tobacco control model. Working with established agencies and leveraging their existing staff, networks, resources, and tools were beneficial from a financial standpoint. However, we also learned that getting buy-in from the staff and frontline workers of the organization is required from the start; in many cases, they become burdened by multiple responsibilities and may not be compensated appropriately. Need-based training of staff for implementing tobacco control measures is also required.

Collaborating with a grassroots organization and leveraging its existing staff and resources was key to successful implementation and sustainability.

Formative research and needs assessment among the villagers, tobacco users, vendors, and workers of grassroots organizations are useful for examining many factors: sociodemographic characteristics, knowledge, attitudes, and levers of change among various community groups; capabilities of frontline workers with respect to delivery of tobacco control interventions; and ways in which vendors can be informed and involved in the TfV process.

Providing alternative income-generating options for vendors who sell tobacco is another critical factor in achieving tobacco-free status in rural areas. Program planners have to include assistance for such stakeholders in the form of financial training or informational support on government schemes for alternative livelihoods. While such measures may convince vendors to switch from selling tobacco to other consumer products, police and legal intervention mechanisms also have to be built into the program. Furthermore, villagers have to be trained in advocacy measures, and provided with timely support and contacts of influential stakeholders who can successfully work with reluctant tobacco vendors who have connections with the local police and political groups.

Interviewers with stakeholders revealed that some of the TfV criteria were difficult to achieve, such as criterion 2, which bans the use of tobacco, and criterion 10, which prohibits visitors from bringing tobacco products into the village. Stakeholders agreed that it was possible that people used tobacco within the private confines of their homes, and residents or visitors purchased tobacco from neighboring areas and smuggled it into the village. There was no mechanism to check if residents or visitors carried tobacco in their bags or pockets when they entered the village. However, stakeholders emphasized that tobacco use in the open public areas visibly stopped due to a change in social norms in tobacco-free villages.

Providing other income-generating options for tobacco vendors is critical in achieving tobacco-free status in rural areas.

Although these 11 TfV criteria were developed through participatory discussions with local stakeholders using the COTPA provisions as a basis, the criteria need to be reexamined with respect to feasibility and accountability. This should occur in the context of a broader dialogue between state and national health ministry officials, international agencies such as WHO, and local village council leaders. Rural tobacco prevention would be well served by meetings that foster dialogue between global, local, and national stakeholders.

## CONCLUSION

Despite national tobacco control policies in India, contextual barriers and lack of proper implementation have enabled tobacco use to persist, especially among rural populations. While tobacco control requires time and dedicated efforts, the TfV program demonstrates that rural tobacco consumption can be controlled and prevented through continuous engagement with village-level stakeholders, motivated local leaders and community workers, contextual and tailored activities, and sanctions and incentives that establish new social norms in villages. This low-cost, community-driven program holds promise for helping public health practitioners and governments implement and achieve the goals of tobacco control policies, especially in resource-scarce settings. Future research, especially in the form of a community trial, is required to further examine program effectiveness. By building on the lessons learned from the TfV intervention, nongovernment and government institutions can ensure better implementation of existing tobacco control policies.

## Notes

**Peer Reviewed**

**First Published Online:** 2017 July 19
